# Use of the movie “Lorenzo’s Oil” for didactic purposes in neuroscience and others health fields

**DOI:** 10.1590/1980-57642020dn14-010002

**Published:** 2020

**Authors:** Lauana Lopes Gonçalves, Tales Alexandre Aversi-Ferreira

**Affiliations:** 1 Universidade Federal de Alfenas Ringgold standard institution – Anatomy. Alfenas, MG, Brazil.

**Keywords:** Lorenzo’s oil, teaching, neurosciences, health field, óleo de Lorenzo, ensino, neurociências, ciências da saúde

## Abstract

Although the traditional method of teaching is still the most popular nowadays, the use of different methodologies such as play approaches, for instance, could be used to make the teaching-learning process a more active approach. Nonetheless, the use of films that represent true stories are more pertinent in active teaching, especially those directly associated with a specific field and that are not merely dramatic. Lorenzo’s oil can inform students about many biological topics and problems linked to intensive care. Furthermore, it also addresses the impact of a neurological disease in a social environment and promotes an intrinsic discussion about sciences in general. Given the above, we propose the hypothesis that the film is useful for educational purposes in health, specifically neuroscience. Lorenzo’s Oil seems to be a good option for the use of a new approach in health science education. The richness of medical topics linked to modern aspects, such as nutrition for patients with mental disorders and palliative care combined with spirituality aspects, promotes an important discussion and constitutes a less stressing learning activity for students. Although some papers cite the importance of the movie for genetics and other fields, this paper shows the importance of efforts to address these topics using a more modern educational approach. According to the results presented, Lorenzo’s Oil could be used extensively for medical/health sciences, confirming the initial hypothesis.

## RESUMO

Embora o método tradicional de ensino ainda seja o mais popular atualmente, o uso de diferentes metodologias, como o lúdico, por exemplo, poderia ser usado para transformar o processo de ensino-aprendizagem em uma abordagem ativa. No entanto, o uso de filmes que representam histórias verdadeiras é mais pertinente no ensino ativo, especialmente aqueles que estão diretamente associados a um campo específico e não são apenas dramáticos. O Óleo de Lorenzo pode informar os alunos sobre tópicos biológicos e problemas relacionados aos cuidados intensivos. Além disso, também aborda o impacto de uma doença neurológica em um ambiente social e promove uma discussão intrínseca sobre as ciências em geral. Diante do exposto, é possível levantar a hipótese da possibilidade de usar o filme em questão para fins educacionais em saúde e especificamente em neurociência. O Óleo de Lorenzo parece ser uma boa opção para o uso de uma nova abordagem na educação em ciências da saúde. A riqueza de tópicos médicos vinculados a aspectos modernos, como nutrição para pacientes com transtornos mentais e cuidados paliativos combinados com aspectos de espiritualidade, promove uma discussão importante e cria uma atividade menos estressante de aprendizado para os alunos. Apesar de alguns trabalhos citarem a importância do filme para a genética e outros campos, este artigo mostra a importância do esforço para adicionar esses tópicos a uma abordagem educacional mais moderna. Então, de acordo com os dados apresentados, o Óleo de Lorenzo poderia ser usado extensivamente para ciências médicas/saúde.

The traditional process of teaching has been based on the educator’s capacity for building and transmitting knowledge via lectures, books and papers,^1^ in this sense, they mirror teachers from previous generations.^2^ Hence, the process of teaching is centered on the teacher and on the retention of information by the students via memorization and, as a consequence, leads to a passive student with a narrow notion of reality.^3^

In fact, many academic fields lack sufficient preparation for teaching, particularly those involving Bachelor degrees.^4^ According to Luckesi,^5^ the traditional methodology of teaching is based on the verbal presentation of a particular subject promoted by the educator using authority, and promoting scant autonomy for students to learn by themselves, an irrational method to be using today.^6^^,^ 7

Many papers claim that the traditional methodologies are flawed because it is assumed that teaching and learning are inseparable, i.e., that teaching is a necessary condition for learning.^1^ Modern theories affirm that learning happens all the time, not necessarily through books and lectures, but also during other activities in life, such as conversations, games and movies. According to cognitive-constructivist theories, the course of learning is a personal construction, which is a result of an experiential process, particular to each person, which translates to a relatively stable behavior modification.^8^ These results can be achieved through the method of active teaching.^4^

Thus, the apprentice is able to learn more effectively if the information is received via various senses, such as auditory, visual, tactile and even kinesthetic,^9^ whereas the learning process arises from the interaction between subject and environment. This method centers on the apprentice as the protagonist of the teaching-learning activities.^8^ Consequently, the apprentice ceases to be a mere spectator and acquires conscience and creativity.^10^^-^ 12

However, sometimes it is not possible to use these modern methods because of the shortage of classes and appropriate materials. Also, there are scant academic studies about new technologies for teaching and learning.^4^^,^ 13 Another problem implementing new technologies in education in Brazil is the reduced time dedicated to subjects in the health area following the reform of the National Curriculum. Therefore, the introduction of these different methodologies is somewhat hampered.^14^ Furthermore, novice teachers tend to feel more insecure about their teaching methods in classes.^15^

 Notwithstanding, despite these difficulties, as an alternative to traditional methodologies, the educator can introduce forms of interactive and experience-based learning methods in a tailored manner using easily accessible resources. For instance, noteworthy examples include the construction of tridimensional embryological models with recyclable materials,^16^ the use of audiovisual resources, such as games and movies during classes,^17^ and other methods previously applied in the health area.^18^^-^ 21 All of these methods, in conjunction, can help students improve their perception of reality.^18^

According to Malik and Agarwal,^17^ the use of multimedia (animations, movies and video games) as an active methodology in several disciplines seems to offer good potential for promoting flexibility, multi-modal and life-long education for learners.

Although the traditional method of teaching is currently still the most popular,^5^ the use of different methodologies, such as the play, could be used to make the teaching-learning process a more active approach. The play epistemology is based on the use of educational games that can be used to improve on traditional ways,^22^ while audiovisual strategies could be used to facilitate teaching in health areas.^23^

Indeed, the active methodology seems to develop many aspects, such as cognitive, emotional, social, economic and cultural in trainees.^3^^,^ 24 ^,^ 25

In this regard, an example that could be studied in the health field is the medical drama “Lorenzo’s Oil” (1992). This popular movie tells the story of a 5-year-old child diagnosed with X-linked adrenoleukodystrophy (ALD), a neurodegenerative disease.^25^^,^ 26 The film is based on a true story and shows how Lorenzo’s parents researched and worked to develop a treatment for their son. In the movie, due to the lack of treatments, the father submits his son to a clinical trial with experimental therapies.

However, Lorenzo did not respond to the treatment and experienced multiple side effects, so his parents withdrew him from the study. Afterwards, his father heard about an experimental treatment, which had not yet been tested on humans, but seemed to be a potentially effective therapy. The parents then contacted the original researcher to organize a meeting to discuss these procedures and produce erucic acid (Lorenzo’s oil) to be tested on their son. The child’s health improves and ultimately the movie gives the feeling that all the parents’ effort was worth it.

However, this kind of movie may cause polemic. For instance, the parents in the movie believed they could develop a treatment for this rare disease by themselves,^28^ a notion which might not sit well with physicians.^27^

Movies are able to disseminate important technological information to the lay population, such as technologies shown in films like Star Trek, Star Wars, and other superhero movies that draw on concepts from quantum and relativity theories, exobiology, spirituality and futuristic technologies. For instance, some of these futuristic technologies have already become reality, such as mobile phones and tablets. However, all of this innovative data contained in movies tends to be undervalued by teachers, who fail to use this excellent technology as support for their classes.^23^

Nonetheless, the use of films that represent true stories are more pertinent in active teaching, especially those that are directly associated with a specific field and are not merely dramatic.^29^ According to Farré,^30^ Lorenzo’s oil (1992) can inform students about biological topics, such as the lipid structure, the dangers of very long saturated fatty acids, the function of cell organelles, inherited diseases and neuroscience concepts, together with problems surrounding the intensive care that people with a severe disability need.^30^ Furthermore, the film also addresses the impact of a neurological disease in a social environment and promotes an intrinsic discussion about sciences in general.^31^

Popular movies are rarely employed during classes about health, compared to social sciences for instance.^30^ Given the above, we propose the hypothesis that the film is useful for educational purposes in health and specifically in neuroscience, particularly for undergraduate students.

## METHODS

The subjects “Lorenzo’s Oil” and “movie” were found in 5 papers on PubMed website, one of which was written in Swedish.

The words “teaching” and “movie” led to the retrieval of 1995 articles on PubMed, but application of the exclusion criteria of papers written in a foreign language, i.e., not written in Portuguese, and papers not focused directly on teaching in Brazil, reduced this total to only 5 articles. On the Scielo database, only one article matched “Lorenzo’s Oil” and “movie”

Reviews, experiences, case reports and academic articles were used in this paper. A few papers, specifically about “Lorenzo’s Oil”, were found, and all of these were utilized.

The inclusion criteria were papers/books providing general data about education that contained the theme “Lorenzo’s oil” linked to “movies” and “teaching”, with an emphasis on studies performed in Brazil. The papers considered relevant for this study had publication dates ranging from 1898 to 2019, spanning Kekule’s history to the present day. For these cases all papers were written in English.

**Figure 1:**
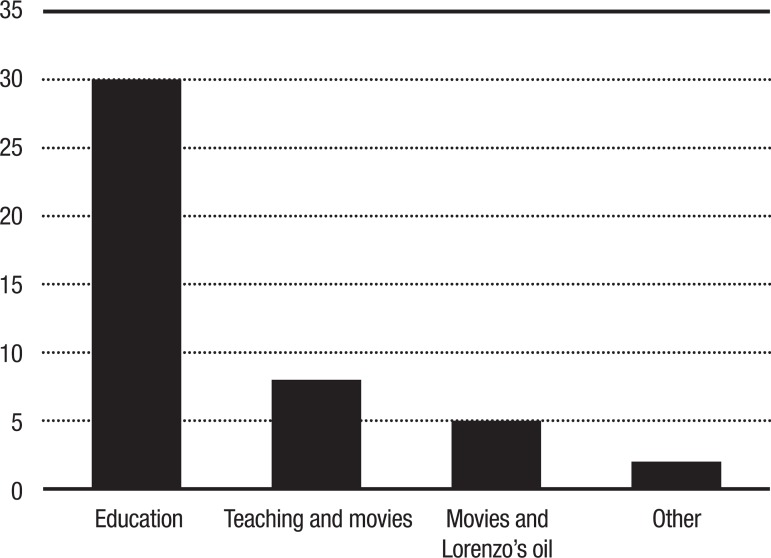
Number of papers/books related to the subject of this review.

Subsequently, papers and books on education were select for this review involving a total of 30 publications, plus 8 associating “teaching” and “movies”, according to the article objective. Another paper on the health science field was also included (Figure 1).

The normality test was rejected because of the small sample size. For the same reason, the Qui-square test was also not used.

## RESULTS

The subjects addressed by the articles and books used in this review are shown in Figure 1. “Education” was the most cited subject, whereas more specifically, the relation between “movies” and “Lorenzo’s Oil” yielded the fewest papers in the search. The term “other” was used for parameters not associated directly with the theme, but linked to interesting related subjects, such as Kekule’s history.

The articles and books on education covered different subjects, such as teaching in many fields e.g. genetics, biochemistry, anatomy, physiology, embryology, neuroanatomy, neuropharmacology and neurophysiology, specifically related to the purpose of this study, namely, the use of Lorenzo’s Oil movie for a teaching objective. Other texts were about general educational theories, including Paulo Freire’s philosophy, *inter alia*.

The papers about teaching and movies mention themes such as clinical pharmacology, neurology and new educational tools. When “Lorenzo’s oil” and “movie” were associated, topics including medicine, genetic, scientific methodology and reactions to this movie were retrieved.

When associating “Lorenzo’s oil movie” plus “teaching subjects”, most papers found were about neurosciences.

Authors that studied the Lorenzo’s oil movie used it for teaching genetics,^31^ medicine in general,^27^ pharmacology^30^ and behaviorism associated with the movie.^26^^,^ 29

The epistemological basis used by these authors plus the considerations regarding active methodologies and new perspectives for teaching were used in this paper.

## DISCUSSION

To date, many studies have been performed worldwide about possible solutions to handle problems of education in many different fields such as mathematics,^32^ engineering,^4^ health sciences and social areas.^33^

Nevertheless, extensive use of new methods for teaching is not observed putatively because of numerous factors and uncontrolled variables.^4^ However, new approaches and methodologies are highly welcome, as are in-depth creative studies about previous suggestions provided in articles and books.

Furthermore, it is important not to see the difficulties implanting new methodologies as an obstacle precluding further studies about new tools for education, i.e., replacing the traditional teaching method characterized by teacher authority and low student autonomy.^5^^-^ 7

Of these concepts, the autonomous process of learning seems to be the most cited theme in articles and books about new educational methods. Given the widely used conservative method of teaching together with the few attempts to implement new methodologies, the concomitant use of both methodologies for a while is suggested. The idea is to gradually introduce new teaching approaches into conservative classes.

New teaching technologies, e.g. audiovisual,^23^ tactile and kinesthetic,^9^ are very important for self-learning.^8^

In this regard, in the implementation of active methodologies, arts and culture could be utilized to foster cognitive, social, economic and emotional maturity in pupils.^3^^,^ 24 ^,^ 25

In fact, culture is a “form of expression and translation of reality”.^34^ Regarded in our culture as the seventh art, the cinema, besides being a cultural and artistic manifestation, must also be analyzed as a means of communication and vehicle for disseminating information.

Thus, in terms of its communicative function, it is possible to observe a powerful educational potential in the new teaching approach,^32^ due to the fact that the cinematographic language often expresses, in an easily understandable way, the several “dimensions of sensitivity, multiple languages and human inventiveness”.^35^

Furthermore, the inclusion of visual arts in teaching has the power to encourage and allow greater critical thinking and can nurture symbolic thinking, a base for superior cognition and learning.^36^ In addition, it can promote reactions linked to aesthetics, provoke the release of feelings, arouse awareness of personal values, stimulate observation and analysis^37^ and simulates experiencing different situations.^38^

Moreover, the use of movies in teaching satisfies the epistemology of new insights in education for the health area. The most acclaimed potential movie for teaching in this area seems to be “Lorenzo’s Oil”.

Notwithstanding the disadvantages that this movie could have for society and physicians,^27^^,^ 28 its importance for teaching is very favorable, considering the opportunity of learning via different means, characterizing a multidisciplinary teaching method.

In terms of health sciences in general and medical information specifically, the movie covers topics that foster comments and studies on behavior and mental diseases for psychiatric discussion at the beginning of the movie.

The relationship between behavior and mental disorders is evident, similar to the initial methods seen in neuropsychology.^39^ The movie shows Lorenzo presenting symptoms like aggressiveness, hyperactivity, violence, motor problems, such as falling off his bike and deafness. However, brain scans disclosed no abnormality.

At the beginning of his recovery, the movements of his eyes and fingers revealed some brain improvement. The cause of deafness was investigated, generating some hypotheses like the presence of a tumor or sclerosis. At this point, the initial concepts about scientific methods were presented and subsequently developed throughout the movie.

Scientific literature searches about adrenoleukodystrophy (ALD), fatty acid metabolism, medical nutrition and the neural system are shown in many parts of the movie. In the literature searched, there was conflicting information about ALD, where Lorenzo’s parents and the scientists were able to resolve some problems by holding a congress to discuss ALD.

A model of the long fatty acid was constructed using clips and, in a dream, Lorenzo’s father realizes how a specific enzyme works. It is a known fact that in science history, dreams can reveal important knowledge. The best known example was the case of Kekule’s dream,^40^^,^ 41 in which he saw a kind of snake swallowing its tail and realized that benzene had a circular structure.

The attempts to achieve a cure were tested and blood exams were used to measure changes in the quantity of fatty acids, a case of hypothesis and responses, utilized in scientific method. Finally, Lorenzo’s father wrote a dissertation about erucic acid after all of his hard work. It is important to point out that medical studies were not his area of expertise and it is plausible to realize the importance of scientific review.

ALD has a genetic origin linked to the X chromosome and the patient’s genealogy. The movie introduces quite basic concepts of medical genetics. In biochemical terms, many concepts were included in the movie, such as oleic acid, unsaturation, enzymatic action, expansion of the carbon chain, the concept of omega fatty acid, erucic acid and 9-omega acid.

In two moments of the film, animal models were regarded as very important for the development of the treatment. This can also be seen in the case of Polish rats and in dogs with myelin problems.

The use of a specific regimen for a neural disorder is extensively shown in the film. A recent approach with nutritional studies associates a close relationship between these two topics.^42^

Symptoms like dysphagia and dysarthria are shown in the movie and could be used in neuroanatomy teaching as a part of the neuroanatomy discipline because of the clinical aspects that are cited, akin to books in this field.^43^

Another important aspect of the movie is the feeling that it provokes in the audience concerning the impact of religiosity and palliative care on a neurologically compromised patient. Lorenzo’s family is deeply religious and the prayers and faith were profoundly demonstrated as well as the mother’s feelings.

Today, religiosity and spirituality have been incorporated into medical care as disciplines in some universities to improve and engender more humanized treatment for patients by medical professionals and students of the health areas.^44^ That is not a recent approach in historical terms, considering that humanity has always been looking for a spiritual cure for diseases.^45^

Among these ideas, new conceptions of teaching in the health field address the medical curriculum, suggesting dialogical ideas for different competencies and integration between theory and practice.^46^ Also, the arts are an implicit subject in education according to Mourthé Junior and colleagues,^47^ especially cinema, the seventh art, because of its approach to rationality and ability to evoke emotions, which are essential in the learning process.^48^ These approaches plus spirituality, support the use of the movie for education in health sciences. An important discussion could be had about palliative care, considering that Lorenzo’s prognosis was death. These types of care were seen primarily from his mother and nurses. In a specific case, a nurse did not accept the duty of reading stories to Lorenzo because of the terminal diagnosis he had been given and because of her medical training.

Adopting new educational methods, a multidisciplinary team could present the film to students and show the cited aspects throughout the movie, pausing at pertinent moments or waiting until the end to engender discussion.

 Teachers from each of the different fields mentioned could use the movie specifically for their discipline, however, the content of the film is also multi/inter and transdisciplinary.

As we get into the new methods compared to the conservative one, Lorenzo’s Oil could be a valuable tool. After watching the movie, reports could be written or a discussion between the teacher and the students about one or more topics presented in the movie could be carried out. Additionally, it is worth pointing out that it is important not to use long periods of time during classes, which is a crucial problem associated with new approaches in education.^4^

The overall purpose of this work in relation to the use of Lorenzo’s Oil movie for didactic purposes is congruent with neuropsychology according to historical and socio-cultural philosophy.^49^ The idea that the environment is necessary for cognitive development^50^^-^ 53 reinforces the need to generate new scenarios for the learning process in all phases of life.

Considering the educational problems outlined in this paper, in order to make suggestions of new approaches and technologies using arts, specifically cinematographic, the bases of neuropsychology were recruited to help indicate ways to improve teaching methods and to make the learning process easier and more attractive.

Incorporating visual and auditory devices in the classroom generates a new and more exciting environment for learning, at least, relative to the usual (conservatory) process of teaching. Furthermore, the cited topics observed in Lorenzo’s Oil permit the initiation of a wide or narrow discussion about neural aspects, depending on the grade of the students, i.e. the period of the undergraduation, promoting a refreshing approach for medical discussions about neuropsychiatry and/or neurological subjects.

## CONCLUSIONS

Given the above, Lorenzo’s Oil seems to be a good option for the use of a new approach in education for health sciences, yet does not restrict the use of conservative teaching. The richness of medical topics linked to modern aspects, such as nutrition for patients with mental disorders and palliative care combined with spirituality aspects, promotes an important discussion and constitutes a less stressing learning activity for students.

Although some papers cite the importance of the movie for genetics and other fields, this paper shows the importance of efforts to address these topics using a more modern educational approach.

Therefore, according to the results presented, Lorenzo’s Oil could be used extensively for medical/health sciences, confirming the initial hypothesis.

## References

[1] Ruben BD (1999). Simulations, games, and experience-based learning: the quest for
a new paradigm for teaching and learning. Simulation Gaming.

[2] Lima SS, Alves FR (2015). Desafios na prática pedagógica do docente iniciante em
instituições de ensino superior. Rev Sab FAMETA.

[3] Vignochi C, Benetti CS, Machado CLB, Manfroi WC (2009). Considerations about problem-based learning in the process of
health education. Rev HCPA.

[4] Alves PA, Aversi-Ferreira TA (2018). Comments on the problems solving methodology in education of
civil engineering in Brazil. RBECT.

[5] Luckesi CC (1999). Procedimentos de ensino. Filosofia da educação.

[6] Lester J, Koehler JR (2003). Fundamentals of information studies: understanding information and its
environment.

[7] Lesh R, Zawojewski JS, Lester F (2007). Problem Solving and Modeling. Second handbook of research on mathematics teaching and
learning.

[8] Aversi-Ferreira TA, Nascimento GNL, Vera I, Lucchese R (2010). The practice of dissection as teaching methodology in anatomy
applied to medical education. Int J Morphol.

[9] Valle LELR (2006). Neuropsicologia e psicopedagogia: desenvolvimento integrado de
competências essenciais para a aprendizagem.

[10] Pietrocola MO (1999). Construction and reality: Mario Bunge's scientific realism and
the teaching of sciences through models. Investigações em Ensinos de Ciências.

[11] Freire P (1979). Educação e mudança.

[12] Freire P (2006). Pedagogia da autonomia: saberes necessários à prática educativa.

[13] Bazzo WA (1996). Qualidade de ensino e sistemas de avaliação.

[14] Castro SKA, Nishijo H, Aversi-Ferreira TA (2018). Neuroanatomy teaching: an example of active teaching applied to
medical formation. Am J Educ Res Rev.

[15] Smaniotto CLD, Gentil VKO (2014). O campo complexo da iniciaçao na docencia.

[16] Freitas LAM, Barroso HFD, Rodrigues HG, Aversi-Ferreira TA (2014). Construction of embryonic models with recycled material for
didactic using. Biosci J.

[17] Malik S, Agarwal A (2012). Use of multimedia as a new educational technology tool a
study. IJIET.

[18] Aversi-Ferreira TA, Monteiro CA, Maia FA, Guimarães APR, Cruz MA (2008). Neurophysiology study associated with three-dimensional models
constructed during the learning. Biosci J.

[19] Araújo J, Galvão G, Marega P, Baptista J, Beber E, Seyfert C (2014). Anatomical challenge: a methodology able to assist in learning
human anatomy. Medicina.

[20] Rocha IRO, Oliveira MHB, Bengston KL, Alves AMN, Brito MVH (2017). Handmade model for peripheral vascular access
training. J Vasc Bras.

[21] Mota MF, Mata FR, Aversi-Ferreira TA (2010). Constructivist pedagogic method used in the teaching of human
anatomy. Int J Morphol.

[22] Castell S (2011). Ludic epistemology: what game-based learning can teach curriculum
studies. JCACS.

[23] Santos SN, Noro A (2013). O uso de filmes como recurso pedagógico no ensino de
neurofarmacologia. Interface.

[24] Melo BC, Sant'ana G (2012). A prática da Metodologia Ativa: compreensão dos discentes
enquanto autores do processo ensino-aprendizagem. Rev CCS.

[25] Berbel NAN (1995). A metodologia da problematização: uma alternativa metodológica
apropriada para o ensino superior. Semina: Cienc Soc Hum.

[26] Moser HW (1999). Suspended Judgement: reactions to the motion picture "Lorenzo's
Oil". Control Clin Trials.

[27] Hudson AJ (2000). Medicine and the movies: Lorenzo's Oil at century›s
end. An Int Med.

[28] Ekins S, Perlstein EO (2018). Doing it all: how families are reshaping rare disease
research. Pharm Res.

[29] Matos FRN, Lima AC, Giesbrecht CM (2011). Estudo observacional das relações de poder no filme O Óleo de
Lorenzo. Cadernos EBAPE.

[30] Farre M, Bosch F, Roset PN, Banos JE (2004). Putting clinical pharmacology in context: the use of popular
movies. J Clin Pharmacol.

[31] Maestrelli SRP, Ferrari N (2006). O Óleo de Lorenzo: o uso do cinema para contextualizar o ensino
de genética e discutir a construção do conhecimento
científico. Genética na Escola.

[32] Nogueira CMI (2016). História da Matemática.

[33] Gomes MPC, Ribeiro VMB, Monteiro DM, Leher EMT, Louzada RCR (2010). The use of active learning methodologies in graduate courses in
health and social sciences: students's evaluation. Ciênc Educ.

[34] Pesavento SJ (2004). História e história cultural.

[35] Teixeira IAC, Lopes JSM (2008). A escola vai ao cinema.

[36] Leontiev NA, Vygotsky LS, Luria AR (2005). Psicologia e pedagogia: bases psicológicas da aprendizagem e do
desenvolvimento.

[37] Camargo CHF (2015). Neurologia e cinema.

[38] Vidal DG (1994). Cinema, laboratórios, ciências físicas e escola
nova. Cad Pesq.

[39] Aversi-Ferreira TA, Watanabe-Tamaiashi BH, Magri MPF, Roqueline AGMF (2019). Neuropsychology of the temporal lobe. Luria's and contemporary
conceptions. Dement Neuropsychol.

[40] Japp FR (1898). Kekulé memorial lecture. J Chem Soc Trans.

[41] Rothenberg A (1995). Creative Cognitive Processes in Kekule's Discovery of the
structure of the benzene molecule. Am J Psychol.

[42] Arsava EM (2017). Nutrition in Neurological disorders: a practical guide.

[43] Machado A, Haertel LM (2006). Neuroanatomia funcional.

[44] Levin J, Schiller P (1987). Is there a religious factor in health?. J Rel Health.

[45] Almeida R (2009). A igreja universal e seus demônios: um estudo etnográfico.

[46] Lima VV (2005). Competência: distintas abordagens e implicações na formação de
profissionais de saúde. Interface.

[47] Mourthe CA, Lima VV, Padilha RQ (2018). Integrating emotions and rationalities for the development of
competence in active learning methodologies. Interface.

[48] Mourthé C (2018). Como a arte cinematográfica e o acesso às emoções contribuem na formação
de profissionais em saúde? SciELO em Perspectiva: Humanas.

[49] Leontiev A (2011). Psicologia e pedagogia: bases psicológicas da educação da aprendizagem e
do desenvolvimento.

[50] Pinheiro M (2007). Fundamentos de Neuropsicologia - o desenvolvimento cerebral da
criança. Vita e Sanitas.

[51] Vygostsk LS (1991). A Formação social da mente: o desenvolvimento dos processos psicológicos
superiores.

[52] Luria AR (1979). The Making of Mind.

[53] Luria AR (1987b). The Man with a Shattered World: The History of a Brain Wound.

